# A Pragmatic Approach to Translating Low- and Very Low-Carbohydrate Diets Into Clinical Practice for Patients With Obesity and Type 2 Diabetes

**DOI:** 10.3389/fnut.2021.682137

**Published:** 2021-07-19

**Authors:** Dina Hafez Griauzde, Kathleen Standafer Lopez, Laura R. Saslow, Caroline R. Richardson

**Affiliations:** ^1^VA Ann Arbor Healthcare System, Ann Arbor, MI, United States; ^2^University of Michigan Medical School, Ann Arbor, MI, United States; ^3^VA Boston Healthcare System, Boston, MA, United States; ^4^University of Michigan School of Nursing, Ann Arbor, MI, United States

**Keywords:** obesity, type 2 diabetes, low-carbohydrate diet, personalized medicine, ketogenic diet

## Abstract

Across all eating patterns, individuals demonstrate marked differences in treatment response; some individuals gain weight and others lose weight with the same approach. Policy makers and research institutions now call for the development and use of personalized nutrition counseling strategies rather than one-size-fits-all dietary recommendations. However, challenges persist in translating some evidence-based eating patterns into the clinical practice due to the persistent notion that certain dietary approaches—regardless of individuals' preferences and health outcomes—are less healthy than others. For example, low- and very low-carbohydrate ketogenic diets (VLCKDs)—commonly defined as 10–26% and <10% total daily energy from carbohydrate, respectively—are recognized as viable lifestyle change options to support weight loss, glycemic control, and reduced medication use. Yet, critics contend that such eating patterns are less healthy and encourage general avoidance rather than patient-centered use. As with all medical treatments, the potential benefits and risks must be considered in the context of patient-centered, outcome-driven care; this is the cornerstone of evidence-based medicine. Thus, the critical challenge is to identify and safely support patients who may prefer and benefit from dietary carbohydrate restriction. In this Perspective, we propose a pragmatic, 4-stepped, outcome-driven approach to help health professionals use carbohydrate-restricted diets as one potential tool for supporting individual patients' weight loss and metabolic health.

## Introduction

Obesity and type 2 diabetes are enduring threats to individual and public health. Dietary habits are generally recognized as key drivers of weight gain and metabolic dysfunction. Yet, the culprit macronutrients and optimal eating patterns for health are fervently debated. Within the U.S., low-fat diets have been the prevailing public health recommendation for chronic disease prevention and management since the 1980s (1). Unfortunately, despite evidence that most U.S. adults embraced low-fat diet ideology (2) and some individuals maintained or restored their health with this approach (3), rates of obesity and related chronic conditions have continued to rise over this time period (4).

Now, amid an obesity epidemic, many recognize that “one-size-fits-all” dietary approaches inherently fail to meet the majority of individuals' diverse metabolic needs, food preferences, and socioeconomic circumstances (5). Policy makers (6) and research institutions (7) have called for the development and use of personalized medicine and nutrition approaches to optimize individuals' health. The National Institutes of Health's 2020–2030 Strategic Plan for Nutrition Research, for example, acknowledges profound inter-individual diet response variation, and urges investigators to explore and address causes of diet treatment effect heterogeneity (8, 9). Additionally, recent clinical practice guidelines for the management of type 2 diabetes (10–12) and obesity (13, 14) acknowledge the potential effectiveness of various dietary approaches for weight loss and glycemic control, which may be recommended and tailored to individual' preferences, needs, and health outcomes.

Despite a theoretical transition from “one-size-fits-all” to personalized dietary approaches, challenges persist in translating some evidence-based eating patterns into the clinical practice. For example, low- and very low-carbohydrate ketogenic diets (VLCKDs)—commonly defined as 10–26% and <10% total daily energy from carbohydrate, respectively—can support weight loss and glycemic control among some individuals while reducing medication use (15–17). Randomized (15, 18) and non-randomized (19–22) clinical trials and real-world observational data (23) also demonstrate favorable changes in high density lipoprotein (HDL) cholesterol, triglycerides, blood pressure, and self-reported measures of energy, hunger, and food cravings. Yet, popular media sources (24) and some nutrition scientists (25, 26) continue to suggest that such eating patterns are less healthy than others and encourage general avoidance rather than judicious use within the context of patient-centered, outcome-driven care.

Critics contend that dietary carbohydrate restriction is too extreme, difficult to sustain over time, and no more effective than other dietary approaches (26). Yet, data demonstrate that this eating pattern can be enjoyable, sustainable, and, more effective for some individuals (27–29). Critics further warn of potential dangers due to high fat intake and a relative dearth of fruits, whole grains, and legumes (30), though the role of dietary fat in disease remains (31), cardiovascular disease risk factors commonly improve with carbohydrate restrictions (32), and high-carbohydrate foods are generally non-essential for human health (33). Some observational studies fuel concerns that low carbohydrate and high fat intake may cause premature death (34); however, such studies cannot completely assess diet quality, degree of carbohydrate restriction, variation in eating patterns over time, objective health measures (e.g., hemoglobin A1c, weight, blood pressure) or health behaviors [e.g., sleep, physical activity; (35)].

While supporters and opponents of dietary carbohydrate restriction have reasonably called for large-scale, long-term randomized-controlled trials to discern the diets' effectiveness and potential risks, this does not obviate the need for pragmatic, real-world strategies to translate current best available evidence and guideline recommendations (10–14) into clinical practice. Moreover, the results of any randomized controlled dietary intervention should only guide—not dictate—treatment for individual patients who may benefit significantly more or less than the average. Across all weight loss interventions, there is marked weight loss treatment heterogeneity (9), and achievement of early weight loss (e.g., within 12 weeks) is a primary predictor of subsequent weight loss and weight loss maintenance (36–43). Accordingly, stepped-care approaches (44–46), adaptive trials (47–50), and N-of-1 studies where individuals serve as their own controls (51) are increasingly recognized as research strategies to discern the appropriate type and intensity of treatment for individuals.

**Figure 1:**
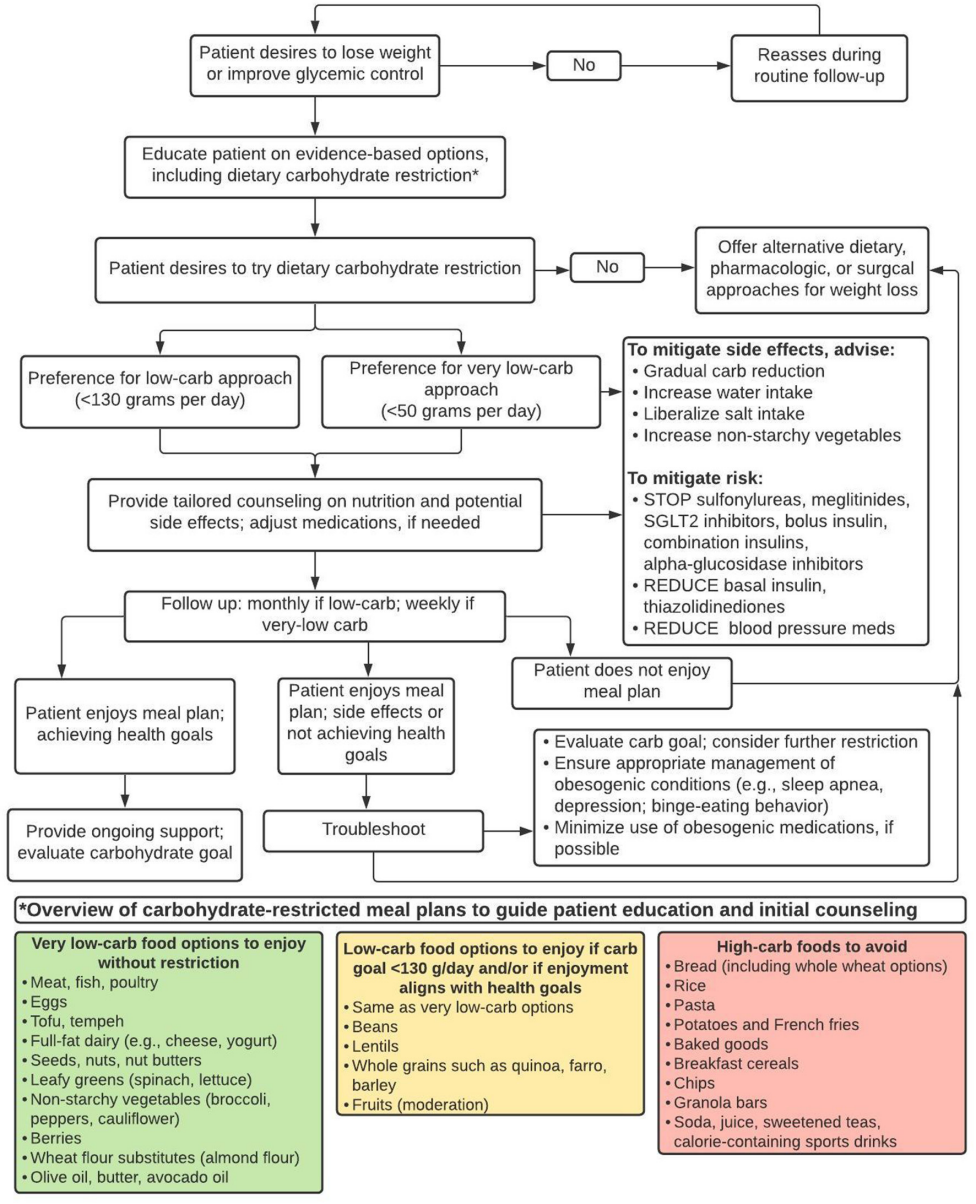
A pragmatic approach to identify and support patients with obesity and/or type 2 diabetes who may prefer and benefit from dietary carbohydrate restriction for weight loss and/or glycemic control.

In clinical practice, however, we already have the opportunity to help individual patients navigate a path to weight loss and improved health by using available evidence-based resources (e.g., lifestyle change programs, medical weight loss programs, bariatric surgery, anti-obesity pharmacotherapy, and nutrition counseling services); recognizing early treatment non-response; addressing barriers; and intensifying or changing treatment plans according to individuals' needs. Carbohydrate-restricted diets are one of many evidence-based treatment approaches. The challenge is to identify individual patients who may prefer and benefit from dietary carbohydrate restriction, and to then use clinical judgment, routine measurements (e.g., weight, blood pressure), periodic laboratory monitoring (e.g., hemoglobin A1c, lipids), and patients' subjective experiences to guide longitudinal care. As with all medical treatments, the potential benefits and risks of specific dietary change must be considered in the context of patients' preferences, needs, and health goals; patients' treatment responses, side effects, and/or adverse reactions should inform subsequent treatment decisions. This is the cornerstone of evidence-based medicine (52). Here we propose a pragmatic, 4-stepped, outcome-driven approach to help providers include low-carbohydrate diets in the menu of options for patients with obesity and type 2 diabetes; this guidance is summarized in Figure 1 and illustrative case examples are provided in the Appendix.

## Step 1: Offer Dietary Carbohydrate Restriction as an Evidence-Based Treatment Option

Providers and patients alike may gravitate toward calorie restriction as the primary path to weight loss. Accordingly, many patients who desire to lose weight have already been advised to follow and have likely tried a calorie-restricted, low-fat diet. While this approach can work for some (3), others may face excessive hunger, food cravings, or non-achievement of health goals despite meticulous dietary adherence (53). Too often, providers respond with persistent recommendations to promote a greater calorie deficit (e.g., drink water before eating, track dietary intake, increase physical activity). Such advice, though well-intentioned, implies patient failure through dietary non-adherence rather than treatment failure and the need for a different approach. Fortunately, clinical practice guidelines for obesity and type 2 diabetes, now support use of alternative eating patterns, such as dietary carbohydrate restriction, which does not require explicit calorie counting and can alleviate excessive hunger and food cravings (53, 54).

## Step 2: Reduce Intake of Common Dietary Culprits of Weight Gain and Metabolic Dysregulation

The degree to which particular individuals should restrict dietary carbohydrate to improve health is relatively untested, and may depend, in part, on their baseline dietary intake and degree of metabolic dysfunction. Some patients, for example, may benefit from general advice to consume, when possible:

- Leafy greens and other non-starchy vegetables (e.g., broccoli) in place of starchy vegetables (e.g., potatoes).
- Whole fruits in place of fruit juice.
- Monounsaturated fats (e.g., olive oil, nuts) as a predominant source of dietary fat.
- Adequate protein (~1 gram/kg body weight/day for most adults).
- Unrefined whole grains (e.g., steel cut oats) in place of refined grains (e.g., breakfast cereal).
- Calorie-free beverages in place of sugar sweetened beverages.
- Limited added sugars.

These recommendations are ubiquitous among seemingly disparate dietary approaches (e.g., low-carbohydrate, plant-based, Mediterranean-style), and may be a reasonable starting point for patients with copious intake of high-carbohydrate foods such as breakfast cereal, granola bars, pasta, starchy vegetables, and/or sugar-sweetened beverages. Protein sources may include eggs, fish, poultry, and/or meat as well as plant-based protein such as tofu, tempeh, beans, and/or lentils. Though inherently more restrictive, low-carbohydrate vegetarian or vegan diets are feasible (55, 56).

Patients' baseline dietary habits, food sources, food budget, and other socioeconomic factors should guide more specific dietary change recommendations. A patient who frequently eats fast food due to long work hours or financial constraints, for example, may be counseled on ways to modify fast food choices (e.g., hamburger wrapped in lettuce instead of on a bun). This may facilitate sustainable lifestyle change and promote physical health benefits without demanding a complete dietary overhaul.

## Step 3: Tailor the Degree of Carbohydrate Restriction to Patients' Preferences and Needs

Patients' change in weight and/or other objective health measures (e.g., glycemic status, blood pressure) should be assessed within 12 weeks of following the recommendations detailed in Step 2. Individuals who do not show early progress toward metabolic goals may need additional support or an alternative treatment (37, 44, 47, 57), which may include tailored dietary advice (58).

Evidence suggests that restricting dietary carbohydrate to ≤ 26% total daily energy (130 grams per 2,000 kcal) may be a reasonable target for many, though some may prefer and achieve greater benefit from further restriction (15). This can be achieved by limiting intake of starches and grains (including whole grains), replacing higher-carbohydrate fruits (e.g., bananas) with lower-carbohydrate fruits (berries), and increasing dietary fat from sources such as avocado, nuts, and full-fat dairy. Patients' subjective experiences and objective outcomes should again be assessed in the near term. Those meeting health goals can be encouraged to continue their current eating pattern.

VLCKDs generally lead to nutritional ketosis when carbohydrate intake is reduced to <10% total daily energy and may be explicitly recommended for patients with type 2 diabetes who desire to reduce medication use or those who do not achieve weight loss and/or glycemic control with more liberal carbohydrate goals; others may also prefer and/or benefit from this as an initial starting point. A well-formulated ketogenic diet includes meats, fish, poultry, eggs, tofu, tempeh, cheese and full-fat dairy, seeds, nuts, leafy greens, non-starchy vegetables, and some fruits (e.g., berries, avocados). Low-carbohydrate wheat flour substitutes such as almond and coconut flours allow individuals to enjoy bread and baked goods while adhering to a ketogenic diet.

Clinical practice guidelines (59) and expert recommendations (16) can guide providers' use of carbohydrate-restricted diets. Websites (e.g., dietdoctor.com, lowcarbprogram.com) and mobile phone applications (e.g., Carb manager, Keto, MyFitnessPal) can support patients, although more intensive programs may be required.

## Step 4: Mitigate Side Effects and Risk

Side effects such as headache, constipation, muscle cramps, and fatigue can occur when transitioning to a VLCKD. A gradual reduction in carbohydrate intake can mitigate symptoms. One practice is to advise patients to change one meal a day per week from high- to low-carbohydrate, starting with breakfast. Snacks, if consumed, may also be changed during the first week. Patients may also be advised to increase their intake of water, salt, and non-starchy, fiber-rich vegetables (16).

Patients who initiate a VLCKD, especially those taking certain medications, should be followed closely to avoid potentials harms. The greatest risks may be hypoglycemia or hypotension due to delays in stopping or reducing anti-hypertensive agents, insulin, and insulin secretagogues. Discontinuation of sodium glucose co-transporter 2 (SGLT2) inhibitors is also recommended to avoid the rare risk of euglycemic ketoacidosis (16).

Dietary carbohydrate—except among those with rare genetic disorders of metabolism—is non-essential for human health (33). Yet, possible micronutrient deficiencies are an inherent risk with all restrictive eating patterns and can be mitigated by emphasizing consumption of low-carbohydrate vegetables and other micronutrient-rich foods (60). A multivitamin can be recommended, if a patient's dietary diversity is limited.

Patients following a VLCKD typically experience favorable changes in triglyceride and high-density lipoprotein (HDL) cholesterol levels (61). Low-density lipoprotein (LDL) cholesterol changes are variable with some patients experiencing marked increased in LDL cholesterol (62). Such elevations may reflect normal fatty acid metabolism (63, 64), and may be less strongly associated with cardiovascular disease risk due to large (vs. small, dense) LDL particles (31). Nevertheless, it is prudent to monitor patients' lipid panel at baseline and within several months of dietary adherence and/or once weight loss has stabilized; unfavorable changes may present an opportunity for shared decision-making and weighing of individuals' potential benefits and risks in the context of scientific uncertainty (62). Patients with relatively mild LDL elevations and improvements in other objective measures (e.g., weight, hemoglobin A1c, blood pressure, triglycerides) and subjective experiences (e.g., increased energy, decreased hunger) may reasonably opt to continue their diet. Others may be counseled to eat lean protein, non-starchy vegetables, and/or gradually liberalize carbohydrate intake, though this approach is based more on anecdote rather than research evidence, which is lacking in this domain (21). A trial of ezetimibe may be considered as diagnostic and therapeutic approach for suspected hyper-absorption of intestinal cholesterol (65).

## Step 5: Support Weight Maintenance

Weight maintenance can be challenging regardless of the initial weight loss approach (66). Patients who lose weight through dietary change, anti-obesity medication use, or weight loss surgery face high rates of weight recidivism (67–69). Certain behaviors commonly support weight maintenance and should be encouraged. These include regular physical activity, reduced sedentary time, routine self-monitoring of weight and/or food intake, adequate sleep, and stress management (66, 70). Occasional dietary indiscretion (e.g., overeating, indulging treats) is inevitable (66). To mitigate feelings of failure and weight regain due to persistent, unrestrained eating behaviors, patients may be encouraged to view “slip-ups” as a natural part of the weight journey and a reminder to actively resume initial weight loss-promoting habits.

Some patients may desire to liberalize their carbohydrate intake when they have reached their health goals. One strategy to help patients identify their individual carbohydrate tolerance threshold may be to increase unrefined carbohydrate intake by ~5–10 gram/day per week (71). If weight gain occurs, patients can be advised to reduce carbohydrate intake to the prior tolerated level.

## Conclusion

The urgent need to effectively confront the global epidemics of obesity and type 2 diabetes is now underscored by the COVID-19 pandemic and its disproportionate harm to individuals with these conditions (72). Across all dietary interventions, there are vast differences in individuals' treatment responses with some individuals gaining weight and others losing weight with the same approach (73–75). Thus, average diet treatment effects and one-size-fits-all nutrition counseling approaches are of little utility when treating individual patients who deserve personalized, outcome-driven care. Using best-available evidence, clinical acumen, and individuals' preferences and needs, we can thoughtfully help our patients navigate a path toward better health with strategies that may include dietary carbohydrate restriction.

## Data Availability Statement

The original contributions presented in the study are included in the article/supplementary material. Further inquiries can be directed to the corresponding author/s.

## Author Contributions

All authors contributed to the drafting and editing of this manuscript. All authors reviewed the submitted draft.

## Conflict of Interest

The authors declare that the research was conducted in the absence of any commercial or financial relationships that could be construed as a potential conflict of interest.
