# Supplementary material for: A Pragmatic Approach to Translating Low- and Very Low-Carbohydrate Diets Into Clinical Practice for Patients With Obesity and Type 2 Diabetes
Source: Front Nutr. 2021 Jul 19;8:682137. doi: 10.3389/fnut.2021.682137 (PMC8326333; doi:10.3389/fnut.2021.682137)
Supplement: Supplementary file 1 [file Data_Sheet_1.docx]

**Appendix:** The three cases below are composites drawn from our clinical experiences with details intentionally changes to protect patient confidentiality. Physical examination findings are normal, unless otherwise noted. The vertical bar (right) includes unpublished qualitative results from our IRB-approved clinical trials using low or very low-carbohydrate diets. We have previously published additional qualitative data from low-carbohydrate diet trial participants (1,2).

*“I've learned that* ***reducing carbs*** *makes a huge difference in how hungry I am. I am pleasantly* ***surprised that I am not hungry*** *between meals.”*

*“I have been obsessing about food every day for years. Eating [a very low-carbohydrate ketogenic] has* ***changed the way I think about food****. Sometimes I even forget to eat because I'm no longer on the blood sugar/insulin rollercoaster and* ***I don't feel hungry 24/7****.”*

*“I have gotten a much better image of myself because I thought I couldn't lose weight because I had no willpower.* ***I was eating little but still not losing.*** *I realized that* ***it wasn't how much I ate as much as what I ate.****”*

*“I would say that* ***understanding the role that insulin and sugar plays in my weight*** *has been incredibly helpful for me. I feel that knowing this will help me continue to stay on track. Some people are lactose intolerant; I feel like* ***I am sugar intolerant*** *in general. Knowing that my body just can't handle sugar helps me avoid it.”*

*“The only thing you're replacing at dinner time from a carb standpoint would be maybe some potatoes or pastas, and there's really great substitutes...There's a low-carb pasta option. And then of course the cauliflower mashed potato kind of thing has always been pretty good...****It really hasn't been a stretch for me at all from a food standpoint.****”*

**Representative Quotes**

**Case 1:** Mrs. C. is a 55-year-old female who presents to primary care clinic for routine follow-up. Her medical history includes type 2 diabetes, obesity, dyslipidemia, hypertension, and obstructive sleep apnea treated with continuous positive airway pressure therapy.

Over the prior 1 year, she has gained approximately 8 kilograms due to frequent fast food meals and convenience store snacks like chips and soda. During the 2 months prior to her clinic visit, she has made efforts to eat healthier. She specifically describes eating oatmeal for breakfast, a banana for snack, and packing a turkey sandwich with a side of pretzels for lunch. She is frustrated that she has not lost weight despite these changes.

Medications include metformin 1000 mg twice daily, glimepiride 4 mg once daily, lisinopril 40 mg once daily, amlodipine 10 mg once daily, and atorvastatin 40 mg once daily.

On examination, blood pressure is 145/86 mm Hg, pulse is 92 beats per minute, weight is 121 kilograms, and body mass index (BMI) is 37 kg/m^2^. She appears fatigued. Laboratory studies are notable for hemoglobin A1c (HbA1c) 9.8%, total cholesterol 311 mg/dL, triglycerides 800 mg/dL, high density lipoprotein (HDL) 29 mg/dL, and low density lipoprotein (LDL) 79 mg/dL.

*Patient-centered treatment plan:* Mrs. C. expresses a strong desire to avoid insulin and other medications that require subcutaneous injection (i.e., glucagon-like peptide-1 receptor agonists). She is offered and is interested in trying dietary carbohydrate-restriction to improve glycemic control and support weight loss. In accordance with clinical practice guidelines for T2DM medication management (3), she is also prescribed empagliflozin 12.5 mg daily. Due to the rare risk of euglycemic ketoacidosis with concomitant use of sodium glucose cotransporter-2 inhibitors and very low-carbohydrate ketogenic diets (defined as <50 grams of carbohydrate per day), Mrs. C is advised to consume a low-carbohydrate diet (defined as 50-130 grams of carbohydrates per day). She is specifically advised to consume a low-carbohydrate breakfast such as eggs in place of oatmeal and to avoid intake of sugar-sweetened beverages.

*8-week follow-up:* Ms. C. is losing weight and reports an increase in energy level. She has achieved 4% body weight loss (weight 116 kilograms, BMI is 36 kg/m^2^) and her HbA1c has decreased by 1.7 points (HbA1c 8.1%). Her lipids also show substantial improvements (total cholesterol 177 mg/dL, triglycerides 293 mg/dL, HDL 40 mg/dL, and LDL 78 mg/dL).

**Case 2:** Mr. M. is a 71-year-old male who presents for routine follow-up. His medical history includes class III obesity (defined as BMI ≥ 40 kg/m^2^), obstructive sleep apnea, non-alcoholic fatty liver disease, hypertension, and congestive heart failure.

Over the prior 15 years, he has gained approximately 65 kilograms, which he attributes to “feeling ravenous.” He consumes primarily home-cooked meals, and his baseline diet consists of cereal, pancakes, home fries, bread, Shepherd’s pie, sandwiches, potatoes, fruit, vegetables, meat, eggs, and milk. During the prior 3 years, he has attempted to make healthier food choices by reading food labels, reducing meat, calories, and salt, and practicing portion control.

Medications include metoprolol 25 mg twice daily, torsemide 100 mg once daily, and valsartan 40 mg once daily.

On examination, blood pressure is 114/64 mm Hg, pulse is 78 beats per minute, weight is 209 kilograms, and BMI is 66 kg/m^2^. He ambulates slowly and there are respiratory crackles at the lung bases bilaterally. He has lower extremity lymphedema.

*“First, I was feeling afraid of fat like, ‘Oh, I better not eat that it's too much fat in it.’ Now I think, like, ‘****Oh the fat is gonna keep me full*** *and it's healthy fat.’ …I'm not shying away from the whole yogurt…now I know I can do whole fat yogurt and it has less sugar.... I just add my... Some berries or something to it…And then I start using avocado oil. And even just adding it to my salad…I started making my own salad dressing. So I know exactly what goes in it. And it gives it that flavor and just* ***experimenting more with recipes*** *and just trying to broaden how I prepare things.”*

*“I’ve lost a large amount of weight.* ***I’m now underneath the threshold of pre-diabetes.*** *Before I started the [very low-carbohydrate diet program] I was massively overweight and* ***I felt that I was going to get diabetes and perhaps not make it.*** *So I’m very thankful [for the program].”*

*“I like feeling in control and I didn't feel that way before. [The]* ***feeling of being in control of my health*** *is very meaningful for me.”*

*“I’ve had a* ***drastic change in my health.*** *I was in the diabetic range; I am no longer in the diabetic range. My cholesterol was high; my cholesterol is not high anymore…My kidney function has not been that great since probably around 2012; now it is normal.”*

**RePresentative Quotes**

Laboratory studies are notable for HbA1c 6.4%, total cholesterol 155 mg/dL, triglycerides 252 mg/dL, HDL 22 mg/dL, LDL 83 mg/dL, creatinine 1.65 mg/dL, and eFGR 42mL/min.

*Patient-centered treatment plan:* Mr. M. is primarily focused on appetite control and achieving and maintaining a weight of 400 pounds (182 kilograms) to improve ambulation and quality of life. He is offered and interested in trying a low-carbohydrate diet (defined as 50-130 grams of carbohydrates per day). Considering his baseline diet, food preferences, and medical history, he is recommended to consume 100 grams of carbohydrates per day. He is advised to consume an egg and vegetable omelet, Greek yogurt, or a protein shake rather than cereal with milk, pancakes, or toast for breakfast. He is provided with a handout including low-carbohydrate meal and snack ideas, and he is provided with monthly nutrition counseling for reinforcement with a registered dietitian over 1 year.

*One-year follow-up:* Mr. M. has achieved 12% body weight loss (weight 184 kilograms, BMI 58 kg/m^2^). He reports a reduced hunger and improved mobility. He no longer has prediabetes (HbA1c 5.4%) and cholesterol levels are improved (total cholesterol 128 mg/dL, triglycerides 133 mg/dL, HDL 25 mg/dL, and LDL 76 mg/dL) His kidney function is stable (creatinine 1.59 mg/dL, eFGR 44mL/min).

**Case 3:** Mr. P. is a 41-year-old male who presents to clinic to discuss weight management. His medical history is significant for type 2 diabetes, obesity, hypertension, and bilateral knee pain. Despite attempting to follow a calorie-restricted diet for the prior 6 months, his weight has increased by 2 kilograms. He states, “it feels like I am starving myself.”

Medications include metformin 1000 mg twice daily, insulin glargine 60 units in the morning and 70 units at night, insulin aspart 10 units three times per day with meals, lisinopril 40 mg once daily, and atorvastatin 80 mg daily.

On examination, blood pressure is 128/75 mm Hg, pulse is 82 beats per minute, weight is 119 kilograms, and BMI is 37 kg/m^2^.

Laboratory studies are notable for HbA1c 11.4%, total cholesterol 166 mg/dL, triglycerides 467 mg/dL, HDL 27 mg/dL, and LDL 86 mg/dL.

*Patient-centered treatment plan:* Mr. P. is willing to make a lifestyle change that will improve his health and reduce his use of medications, particularly insulin. He is offered and interested in trying a very low-carbohydrate diet (<50 grams of carbohydrate per day) with close monitoring by a clinical pharmacist to adjust medications. He is also offered and agrees to use a continuous glucose monitor to facilitate timely medication changes and avoid hypoglycemia. He meets once weekly for four weeks with a dietitian who helps him transition from a high- to a very low-carbohydrate eating pattern by changing one meal per week, starting with breakfast and snacks.

*12-week follow-up:* Mr. P. states “I feel better than I have in 10 years.” He is no longer requiring insulin aspart and insulin glargine has been reduced to 53 units once daily. He has achieved 7.6% body weight loss (weight 110 kilograms, BMI is 32 kg/m^2^) and his HbA1c has decreased by 2.9 points (HbA1c 8.5%). His lipids also show favorable changes (total cholesterol 145 mg/dL, triglycerides 212 mg/dL, HDL 28 mg/dL, and LDL 75 mg/dL).

1. Griauzde DH, Saslow L, Patterson K, Ansari T, Liestenfeltz B, Tisack A, Bihn P, Shopinski S, Richardson CR. Mixed methods pilot study of a low-carbohydrate diabetes prevention programme among adults with pre-diabetes in the USA. *BMJ Open* (2020) **10**:e033397. doi:10.1136/bmjopen-2019-033397

2. Yost O, DeJonckheere M, Stonebraker S, Ling G, Buis L, Pop-Busui R, Kim N, Mizokami-Stout K, Richardson C. Continuous Glucose Monitoring With Low-Carbohydrate Diet Coaching in Adults With Prediabetes: Mixed Methods Pilot Study. *JMIR Diabetes* (2020) **5**:e21551. doi:10.2196/21551

3. Association AD. 9. Pharmacologic Approaches to Glycemic Treatment: Standards of Medical Care in Diabetes—2020. *Diabetes Care* (2020) **43**:S98–S110. doi:10.2337/dc20-S009
